# Proteomic Landscapes of 3D and 2D Models of High-Grade Serous Ovarian Carcinoma: Implications for Carboplatin Response

**DOI:** 10.1021/acs.jproteome.5c00391

**Published:** 2025-08-27

**Authors:** Jimmy Maillard, Theodoros I. Roumeliotis, Ekta Paranjape, Lisa Pickard, Alvaro Ingles R. Garces, Jyoti S. Choudhary, Udai Banerji

**Affiliations:** † Clinical Pharmacology Adaptive Therapy group, Division of Clinical Studies and Division of Cancer Therapeutics, 117534 Institute of Cancer Research, London SM2 5NG, United Kingdom; ‡ Functional Proteomics Group, Chester Beatty Laboratories, 5053 The Institute of Cancer Research, London SW3 6JB, United Kingdom; § The Drug Development Unit, The Institute of Cancer Research and The Royal Marsden Hospital NHS Foundation Trust, London SM2 5NG, United Kingdom

**Keywords:** 2D cell culture, 3D cell culture, ovarian cancer, whole cell proteomics

## Abstract

High-grade serous ovarian carcinoma (HGSOC) is the most common form of ovarian cancer, and finding new treatments remains an unmet need. While drug discovery is typically performed in two-dimensional (2D) monolayers, three-dimensional (3D) culture systems better mimic the in vivo conditions. However, a comprehensive comparison of 3D versus 2D ovarian cancer models is lacking. Here, we quantitatively compared the whole cell proteomic signatures of four ovarian cell lines—PEO1, PEO4, UWB1.289, and UWB1.289+BRCA1—with different status of BRCA genes grown in 2D and 3D. Using isobaric labeling proteomics, we quantified 6404 proteins and identified 371 significantly and commonly altered proteins between 2D and 3D. Proteins upregulated in 3D were enriched for transmembrane transport and NADH:ubiquinone oxidoreductase complex I, while energy metabolism and cell growth pathways also showed dimensionality-dependent changes. Notably, membrane-associated proteins were downregulated in spheroids, particularly EGFR in PEO1. Furthermore, the 3D culture modulated the response to carboplatin, with an increased expression of drug resistance-associated proteins, including NDUF family members in all spheroid models. These findings underscore how culture dimensionality influences both the molecular landscape and the chemotherapeutic response of HGSOC cells and highlights candidate targets for overcoming carboplatin resistance.

## Graphical abstract

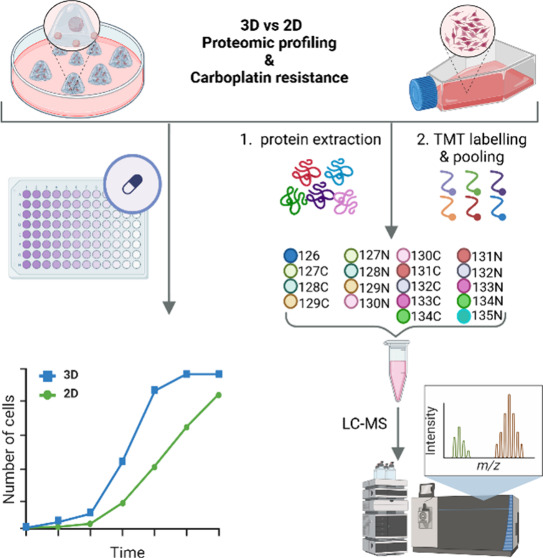

## Introduction

Ovarian cancer is the leading cause of mortality among gynecological malignancies, with over 300,000 cases diagnosed and over 200,000 deaths reported worldwide every year.^1,2^ High-grade serous ovarian carcinoma is the most common form of ovarian cancer, and patients are often diagnosed with metastatic disease and cannot be cured.^3^ While finding new treatments is an unmet need, immuno- and targeted therapies targeting surface proteins are promising future therapeutic approaches.^4^ Membrane-related cancer biomarker identification, a pivotal step in the development of such approaches,^5^ is mostly performed on two-dimensional (2D) cell monolayers.^6–8^ Limitations of these approaches include the fact that 2D models do not reproduce complex in vivo cell–cell and cell-extracellular matrix interactions^9^ which might affect the expression of membrane-related proteins. Therefore, in vitro cellular models that more accurately reflect in vivo conditions are needed.

Over the last 2 decades, spheroids have gained interest as three-dimensional (3D) models for preclinical in vitro studies due to the ease of implementation and better representation of in vivo-like cell–cell and cell-matrix interactions than 2D cell culture.^10–13^

Quantitative MS-based methods have been successfully used to profile changes in the cellular proteome from different types of 2D and 3D cultured cells, including colorectal,^14–16^ glioblastoma,^17^ breast,^18^ cervical,^19^ and lung^20^ cancer cell lines, as well as fibroblasts and cancer-associated fibroblasts.^21^ These whole cell proteomics and phosphoproteomics studies commonly identified upregulation of proteins associated with energy metabolism and downregulation of proteins involved in DNA regulation and cell proliferation in 3D models.

Recently, Kerslake et al. performed a meta-analysis and experimental comparison of the transcriptomic variations in 3D vs 2D ovarian cancer models.^22^ They reported that glycolysis, KRAS signaling, oxidative phosphorylation, and TNF-α signaling via NF-κB are typically differentially regulated at the transcriptome level in ovarian cell lines depending on the cell culture dimension.^22^

Till date, a thorough description of the proteomic landscape that captures the post-transcriptional and post-translational regulation^23,24^ of 3D vs 2D high-grade serous ovarian carcinoma (HGSOC) cancer models is missing. Here, we provide an insight into the proteomic characteristics of 3D spheroids; we compared the whole cell proteomics signature of four HGSOC cancer cell lines (PEO1, PEO4, and UWB1.289, UWB1.289+BRCA1) grown in 2D and in 3D. PEO1 and PEO4 cells were isolated from the same patient, diagnosed with HGSOC, before and after developing resistance to platinum-based chemotherapy and is known to harbor the *BRCA2* mutation.^25^ UWB1.289+BRCA1 (in which the BRCA1 gene is restored) is a genetically modified version of the UWB1.289 HGSOC cell line, which originally carries a BRCA1 mutation.^26^ These genomically similar cell line pairs are ideal to study differences in the proteome caused by culture conditions and to investigate differences in drug sensitivity.

We aimed to study the differences in whole cell protein expression in ovarian cell lines and predict the membrane protein expression between 3D and 2D culture conditions. We found that proteins involved in DNA regulation and energy metabolism are significantly differentially regulated, depending on the cell culture dimension. We also investigated whether the culture conditions invoked any functional therapeutic outcomes like carboplatin resistance.

## Experimental Procedures

### 2D and 3D Cell Culture

UWB1.289 (ATCC) and UWB1.289+BRCA1 cells (ATCC) were maintained in complete RPMI 1640 (ThermoFisher Scientific) supplemented with 2 nM l-glutamine (Life technologies), 10% fetal bovine serum (PanBio Supreme P30-3031), 1× MEM nonessential amino acid solution (Sigma-Aldrich) as base media to which is also added 5 μg/mL insulin (Sigma-Aldrich), 10 ng/mL hEGF (Thermo Fisher Scientific), and 500 ng/mL hydrocortisone (Sigma-Aldrich) diluted from a 1 mg/mL stock solution prepared in 70% ethanol and stored at −20 °C. PEO1 (ECACC) and PEO4 (ECACC) were cultured using the same base media but supplemented with 1 mM sodium pyruvate (Thermo Fisher Scientific).

All cells were cultured in T175 flasks (Thermo Fisher Scientific) for 2D cell culture and harvested using 0.05% trypsin-EDTA (Sigma-Aldrich). To generate spheroids, cells were harvested from 2D cell monolayers and resuspended in cold (4 °C) Corning matrigel growth factor reduced basement membrane matrix (Corning Life Sciences), referred to as Matrigel here, at a concentration of 400,000 cells/mL. 200 μL domes were deposited at the bottom of the wells of 12-well plates. Matrix-embedded cells were incubated for 30 min at 37 °C before adding 2 mL per well of their respective 2D cell culture media. Spheroids were allowed to form for 11 days, and media was changed twice a week. 2D and 3D cells were maintained in a humidified incubator at 37 °C in 5% CO_2_. Owing to a very low spheroid recovery yield from Matrigel domes using commercial harvesting solutions, spheroids were harvested with a custom-made harvesting solution containing 15% (v/v) ethanol (Sigma-Aldrich), 2.5% (w/v) 1,6-hexanediol (Sigma-Aldrich), glycerol 6% (v/v) (Thermo Fisher Scientific), 0.5% (w/v) 2-hydroxethyl cellulose (Thermo Fisher Scientific), 0.5% (w/v) carboxmethyl cellulose (Thermo Fisher Scientific), and 150 mM d-glucose (Thermo Fisher Scientific) prepared in phosphate-buffered saline (PBS) (Gibco).

### Western Blot Analysis

For the Western blot (WB) analysis, cells and spheroids were cultured and harvested as described in the previous section. Cells were lysed in RIPA buffer supplemented with protease and phosphatase inhibitors (mini cOmplete protease inhibitor cocktail, Roche), and proteins were quantified using a BCA assay (Thermo Fisher Scientific). Thirty μg of protein was loaded and separated using 4–12% Bis-Tris gel electrophoresis (Invitrogen). Separated proteins were transferred onto nitrocellulose membranes using an iBlot 3 WB transfer system (Thermo Fisher Scientific). Membranes were blocked (BSA 3%) and incubated at room temperature with an anti-GAPDH (1:2000, Cell signaling technologies) antibody for 2 h as the loading control. Membranes were then incubated at room temperature for 1 h with AF680-conjugated secondary antibodies (1:10,000, LI-COR Biosciences), and fluorescent signals were imaged on an Odyssey XF Imager. Membranes were then stripped and incubated overnight at 4 °C with an anti-EGFR (1:1000, Cell signaling technologies) antibody. Fluorescence labeling and imaging of EGFR were performed similarly for GAPDH.

### Carboplatin Drug Treatment in 3D and 2D Cell Cultures

Cell viability following carboplatin (Apex-Bio) treatment in 2D cell cultures was determined by using a CellTiter-blue growth assay (Promega). PEO1, PEO4, UWB1.289, and UWB1.289+BRCA1 were plated at 4000, 3000, 2000, and 2000 cells/well in the wells of a 96-well plate (Sigma-Aldrich). On the day after plating, media was changed, and cells were incubated with various concentrations of carboplatin (0–300 μM) for 5 days. Fresh carboplatin stock solutions (20 mM) were prepared in 0.9% NaCl solutions, and dilutions were prepared in the respective media of each cell lines. For fluorescence measurements, media was removed, and 100 μL of fresh media was added with 10 μL of CellTiter-Blue reagent (Promega). Fluorescence intensity was measured after 3 h of incubation at 37 °C at 590 ± 10 nm using a plate reader (PerkinElmer Victor *X*4). Excitation was provided at 531 nm. A similar analysis was conducted on 3D cultured cells with the exception that cells were plated in 50 μL Matrigel domes, and spheroids were allowed to form for 3 days (using the same initial cell count per well as for 2D cells) before drug exposure. Fluorescence background corrections and GI_50_ (growth inhibition) calculations were performed using GraphPad Prism10 based on three independent measurements.

### Sample Preparation for LC–MS Analysis

After harvesting, 2D and 3D cells were washed three times in cold PBS, pelleted (∼5 million cells per pellet), and stored at −80**°**C. Cell pellets were lysed in a buffer containing 1% sodium deoxycholate (SDC), 100 mM triethylammonium bicarbonate (TEAB), 10% isopropanol, and 50 mM NaCl, freshly supplemented with 5 mM TCEP (Thermo, Bond-breaker), 10 mM iodoacetamide (IAA), universal nuclease 1:2000 vol/vol (Pierce, #88700), and Halt protease, and phosphatase inhibitor cocktail (Thermo, #78442, 100X) with 5 min of bath sonication and 30 s of probe sonication. Protein concentration was measured with the Quick Start Bradford protein assay (Bio-Rad). Three reference samples were prepared by randomly grouping the samples into three sets and pooling equal protein amounts from each sample within a set. Aliquots of 30 μg of total protein were digested overnight with trypsin (Pierce, 1:20) at room temperature. Peptides were labeled with the TMTpro reagents (Thermo) by adding 5 μL of the reagent (25 μg/μL) into 12.5 μL of sample volume. Samples were randomly assigned to four TMTpro batches with all three reference samples included in each batch to enable cross-batch normalization (Supplementary file S1). The TMTpro mixture was acidified with formic acid at 2%, and the precipitated SDC was removed by centrifugation. The peptide pool was fractionated with high pH reversed-phase chromatography using the XBridge C18 column (2.1 × 150 mm, 3.5 μm, Waters) on an UltiMate 3000 HPLC system over a 1% gradient in 35 min. Mobile phase A was 0.1% (v/v) ammonium hydroxide, and mobile phase B was 0.1% ammonium hydroxide (v/v) in acetonitrile. To assess potential contamination from Matrigel constituents, mock samples consisting of 40 or 200 μL of Matrigel domes (three of each) were prepared without cells in 1.5 mL of Lo-bind Eppendorf tubes and processed using the same workflows as for 3D samples (six samples). Contaminant proteins were solubilized in 100 μL of 100 mM TEAB buffer, reduced and alkylated with 5 mM TCEP and 10 mM IAA, respectively, and digested overnight with 50 ng/μL trypsin concentration. Peptides were SpeedVac dried and subjected to label-free LC–MS/MS analysis. For the data-independent acquisition (DIA) analysis, cell pellets were lysed and digested as described above for the TMT analysis, and SDC was precipitated in each individual sample with acidification followed by centrifugation. Peptides were SpeedVac dried and reconstituted in 0.1% TFA at 200 ng/μL for DIA analysis.

### LC–MS Analysis and Data Processing

LC–MS analysis was performed on an UltiMate 3000 system coupled with the Orbitrap Ascend mass spectrometer (Thermo) using a 25 cm capillary column (Waters, nanoE MZ PST BEH130 C18, 1.7 μm, 75 μm × 250 mm) over a 100 min gradient 5–27% mobile phase B composed of 80% acetonitrile and 0.1% formic acid. Peptides were preconcentrated onto an Acclaim PepMap 100, 100 μm × 2 cm C18, 5 μm trapping column at 10 μL/min of 0.1% TFA, and the analytical column was connected to an EASY-Spray emitter (Thermo ES991). MS spectra were collected at Orbitrap mass resolution of 120k, and precursors were targeted for HCD fragmentation in the top speed mode (3 s) with a collision energy of 32% and iontrap detection in turbo scan rate. MS3 scans were triggered by real-time search (RTS) against a fasta file containing UniProt *Homo sapiens* reviewed canonical and isoform sequences with multinotch isolation (10 notches) and HCD fragmentation with collision energy 55% at 45 K Orbitrap resolution. Targeted precursors were dynamically excluded from further activation for 45 s with 10 ppm mass tolerance, and RTS close-out was enabled with maximum four peptides per protein. Static modifications for RTS were TMTpro16plex at K/N-term (+304.2071) and carbamidomethyl at C (+57.0215), and variable modifications were deamidated NQ (+0.984) and oxidation of M (+15.9949) with a maximum of one missed cleavage and two variable modifications per peptide.

The Matrigel background digests were analyzed on a Vanquish Neo HPLC system coupled with an Orbitrap Ascend mass spectrometer (Thermo) using an 80 min gradient and HCD-MS2 method with CE 32% at 45 K Orbitrap resolution. The six raw data files were processed with the Sequest HT node in Proteome Discoverer 3.0 (Thermo) for protein identification against a fasta file containing reviewed *Mus musculus* protein sequences. This analysis identified 846 mouse protein groups that were considered as contaminants in the analysis of the human cells cultured in 3D as described below.

The Sequest HT and Comet nodes in Proteome Discoverer 3.0 (Thermo) were used to search the raw mass spectra against a fasta file containing reviewed UniProt *Homo sapiens* entries concatenated with the 846 contaminant Matrigel mouse proteins identified in the mock extraction analysis. The precursor mass tolerance was set at 20 ppm, and the fragment ion mass tolerance was set at 0.5 Da (or 1 Da for Comet) with up to two trypsin missed cleavages allowed. TMTpro at the N-terminus/K and carbamidomethyl at C were defined as static modifications. Dynamic modifications were oxidation of M and deamidation of N/Q. Peptide confidence was estimated with the percolator node, and peptide FDR was set at 0.01 based on target-decoy search. The Reporter Ions Quantifier node in the consensus workflow included the following settings to ensure the use of peptides uniquely matching human or mouse proteins: Peptides to Use = Unique, Consider Protein Groups for Peptide Uniqueness = False, and Use Shared Quan Results = False. Peptides with average reporter signal-to-noise greater than 3 were used for protein quantification. All identified mouse proteins were eventually filtered out from the downstream analysis.

DIA analysis was performed with an 80 min gradient 3–27% phase B for 2 μg of peptide loading per sample. MS1 spectra were collected with a mass resolution of 60 K in the *m*/*z* range of 380–985, with the maximum injection time 100 ms and AGC 4 × 10̂5. DIA MS2 spectra were collected with HCD fragmentation CE 32%, orbitrap resolution 15 K with isolation window 10 and 1 *m*/*z* overlap, maximum injection time 40 ms, and AGC 1 × 10̂5. Raw data were processed in the DIA-NN software version 2.0.2 for protein identification and quantification in the library-free mode using a fasta file containing reviewed UniProt human proteins concatenated with the 846 contaminant Matrigel mouse proteins. Carbamidomethylation of C and oxidation of M were defined as fixed and variable modifications, respectively. MBR was enabled, and proteins were filtered at 1% FDR. Protein groups containing at least one accession number from the mouse proteins were excluded from further statistical analysis and comparison with the TMT data.

### Statistical Data Analysis

Statistical data analysis was performed with an in-house pipeline in RStudio. Raw protein-level signal-to-noise values were exported from Proteome Discoverer, and only Master proteins with at least one nonzero value across samples within a TMTpro set were retained for further analysis. Proteins with missing values (NAs) across an entire TMT batch were excluded from analysis, whereas proteins containing one or more zero values within a batch were retained as zeros likely reflect true biological absence rather than technical failure in the multiplexed data. Zero values (representing <0.5% of the data set) were replaced with a minimum value of 0.1 to enable ratio generation, followed by sample-wise median normalization and log_2_ transformation. An initial principal component analysis (PCA) plot, generated after subtracting the row mean across samples to evaluate batch effects, confirmed the necessity of using the reference samples for normalization. Based on the latter, the log_2_-transformed data were further scaled by subtraction of the mean of the reference samples per TMT set, and data were finally centered at zero across all samples from the different TMT sets. Differential analysis was performed using the row.oneway.anova function from the Bioconductor HybridMTest library (Pounds S, Fofana D, 2022) for comparisons involving two or more sample groups. Over-representation and GSEA analysis and visualization of Gene Ontology (GO) terms were performed with the clusterProfiler library.^27^ GO annotations were downloaded from UniProt. The ggplot2, gplots, ggridges, ComplexHeatmap, and circlize packages were used for generation of plots. Specifically R version 4.2.0 was used with the following packages versions: AnnotationDbi (1.60.2), Biobase (2.58.0), BiocGenerics (0.44.0), circlize (0.4.16), clusterProfiler (4.6.2), ComplexHeatmap (2.14.0), dplyr (1.1.1), fdrtool (1.2.17), ggplot2 (3.5.0), ggrepel (0.9.5), ggridges (0.5.6), gplots (3.1.3.1), HybridMTest (1.42.0), org.Hs.eg.db (3.16.0), pdftools (3.4.0), purrr (1.0.2), qpdf (1.3.3), readr (2.1.4), reshape2 (1.4.4), stringr (1.5.0), tidyr (1.3.0), and tidyverse (2.0.0).

Membrane protein annotations were downloaded from the Human Protein Atlas using the following filter: subcell_location:Plasma membrane,Cell Junctions;Enhanced,Supported. For analyses involving membrane-associated protein annotations, *p*-values lower than 0.05 were considered to apply less stringent thresholds to minimize exclusion of potentially relevant changes.

## Results

### Global Differential Analysis of 3D and 2D Cultured HGSOC Cells

Global proteome characterization to elucidate quantitative differential expression levels between 3D and 2D cultures of PEO1, PEO4, UWB1.289, and UWB1.289+BRCA1 cells were conducted using a TMT-labeling strategy. Specifically, samples were run in triplicate and spread across multiple TMTpro 18-plex sets. After reduction, alkylation, trypsin digestion, TMT labeling, and sample pooling, the pooled peptides were fractionated off-line on a high pH reversed-phase column for data-dependent acquisition with RTS MS3 quantification for high accuracy and precision. We quantified 6404 proteins across all samples without missing values, demonstrating deep proteome coverage.

First, we generated an initial PCA plot without applying reference-based normalization, which revealed batch effects and poor clustering of the replicate reference samples (Figure S1). This observation supports the necessity of the reference-based normalization strategy used in our downstream analyses. After normalization, we generated a pairwise correlation matrix to highlight similarities and differences in protein expression patterns across all four cell lines grown in 2D and 3D (Figure 1a). Notably, clusters of high correlation are observed between PEO1 and PEO4 cells and between UWB1.289 and UWB1.289+BRCA1 cells; however, the greatest similarity in the proteome was driven by the culture condition rather than the closely related genomics of the cell line pairs (Figure 1a). Further, 2D PCA highlights sample clustering based on cell identity (Figure 1b) and culture dimension as the main sources of proteome variation (Figure 1c). Across-18plexes replicates of the reference samples tightly cluster together, demonstrating efficient correction of batch effects and high reproducibility of the data (Figure 1b,c).

**Figure 1:**
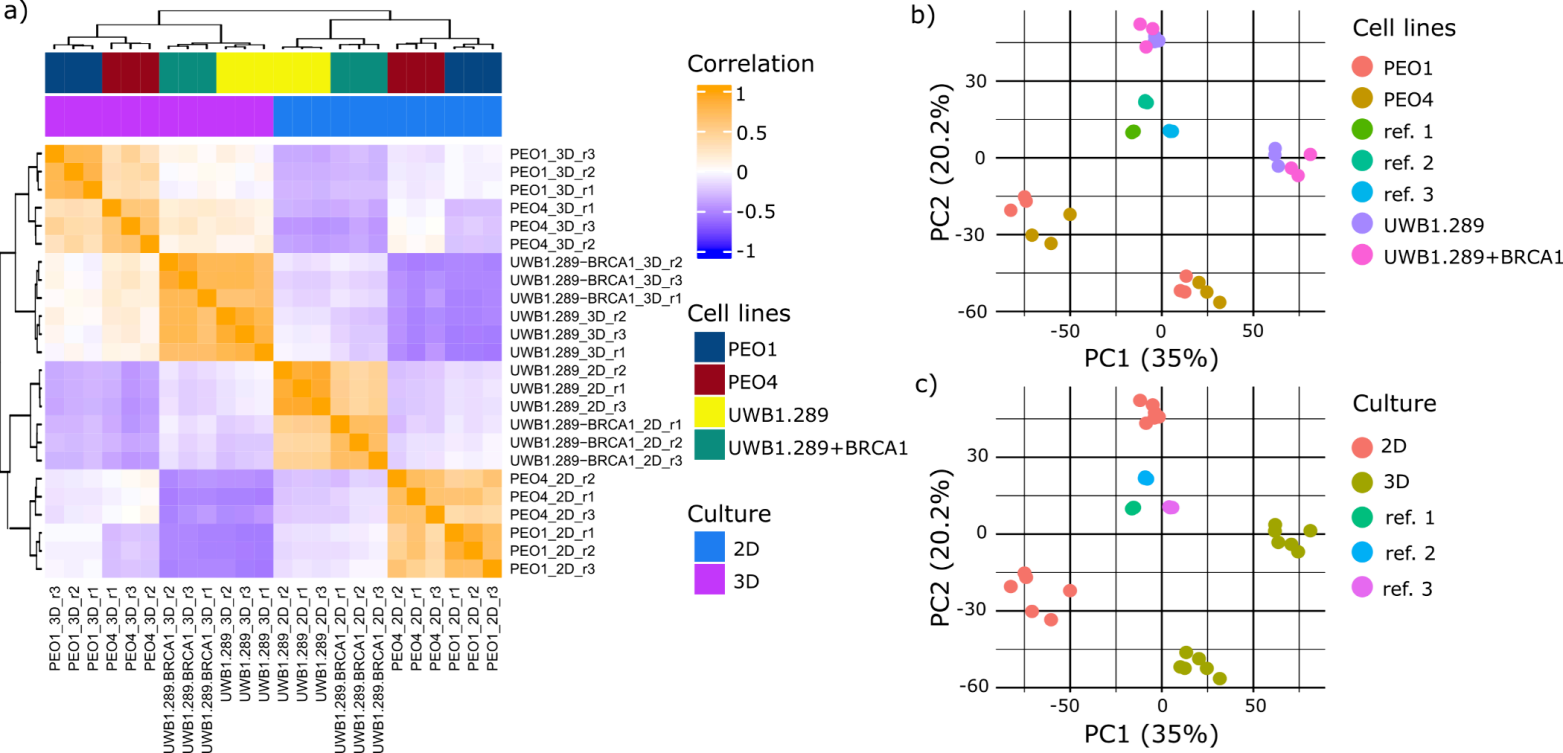
Unsupervised sample clustering. (a) Correlation matrix of HGSOC biological models grown in 2D and 3D based on their overall protein content; samples were run in triplicates. (b, c) 2D PCA score plots of the entire data set, respectively, highlighting cell type and culture dimension-dependent clustering.

To better understand the biological processes associated with the global proteome variation, we then determined statistically significant differences in protein abundance across different sample groups for each cell line and culture dimension from triplicate experiments using ANOVA (Supplementary file S2). We visualized reproducible differences in protein abundance in a heatmap (Figure 2a) using relative log_2_ ratio to the mean of each protein across all samples (thresholds; ANOVA *p*-value < 0.01 and top 30% most variable by standard deviation), which revealed 1680 proteins with reproducible differential expression between cell lines and culturing conditions.

**Figure 2:**
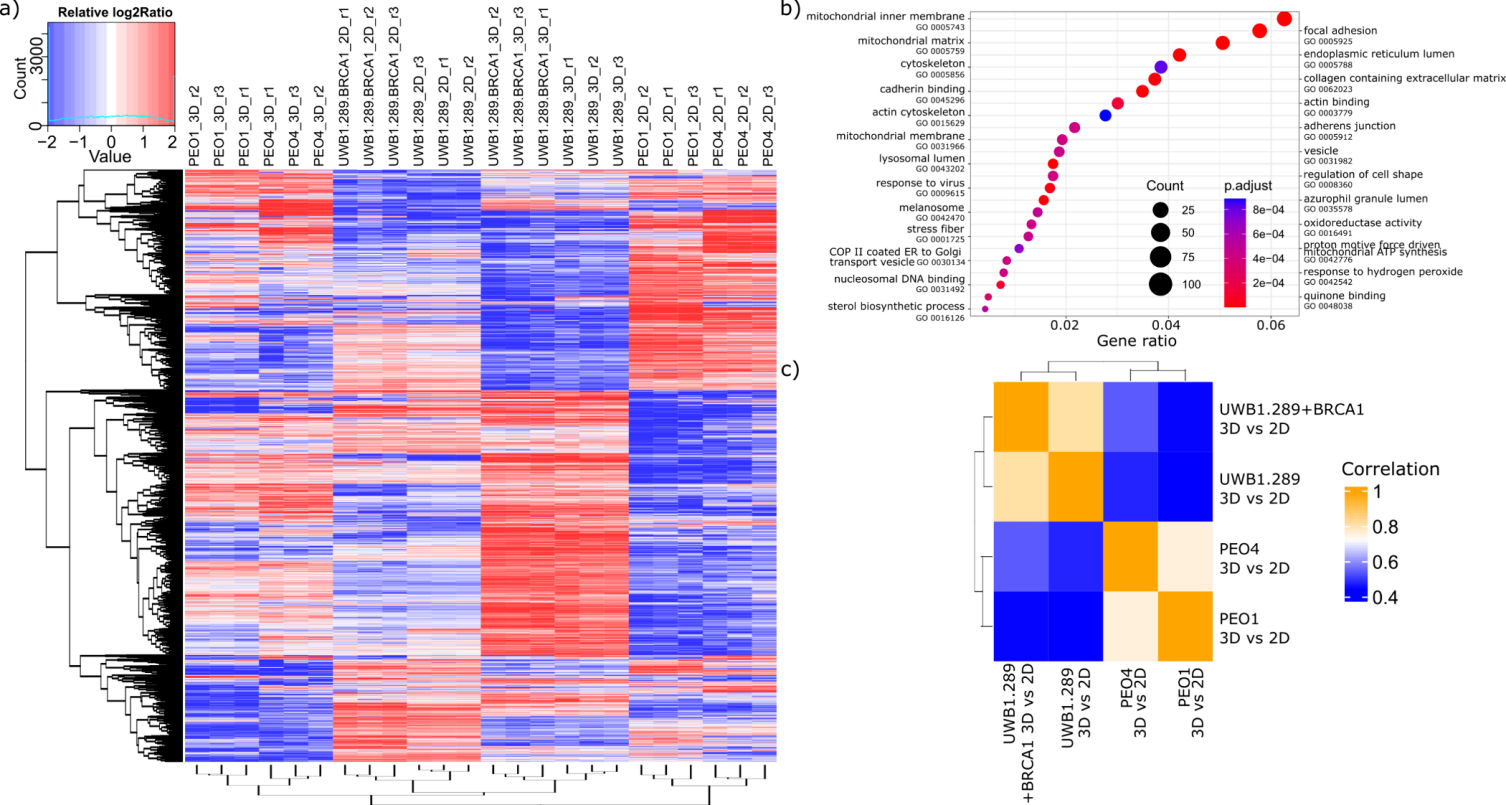
Biological processes associated with proteome variation and similarity of 3D and 2D comparisons between cell lines. (a) Heatmap of the most differentially abundant proteins in PEO1, PEO4, UWB1.289, and UWB1.289+BRCA1 cells in 2D and 3D samples run in triplicate experiments. Data filtering was performed with ANOVA *p*-value < 0.01 and top 30% most variable by standard deviation. (b) Dot plot showing the over-representation analysis performed on the data set presented in panel (a) using GO terms. (c) Correlation matrix comparing the log_2_ ratio of 3D versus 2D proteomes of each cell line.

We then performed an over-representation analysis on this set of proteins using GO terms to identify differentially regulated biological processes (Figure 2b). We found that proteins most differentially expressed among all conditions are primarily involved in energy metabolism, with enriched terms such as oxidoreductase activity, mitochondrial inner membrane, and cell–cell interactions, and membrane-associated proteins with terms such as focal adhesion and cadherin binding (Figure 2b).

To assess whether the 3D versus 2D proteome differences observed for each cell line are conserved across cell lines, we performed correlation analysis using the log2ratio of 3D versus 2D of all proteins for each cell line (Figure 2c). This analysis showed that culture dimension very distinctively affects UWBs (UWB1.289 and UWB1.289+BRCA1) and PEOs (PEO1 and PEO4) cells (Figure 2c), but with higher, yet very moderate, similarity between cell lines with closely related genotypes.

### Protein Expression Changes Attributed to Culture Conditions

To interrogate the differential protein abundance effects of 3D models in detail, we next performed comparative analysis for each cell line grown in 3D and 2D, with a *p*-value threshold of 0.01 and an absolute log_2_ fold change > 1 (Figure 3and Supplementary file S3). Under these conditions, we identified 1884 differentially regulated proteins in PEO1 cells (Figure 3a) between 3D and 2D culture settings, 1329 proteins in PEO4 cells (Figure 3b), 2114 proteins in UWB1.289 cells (Figure 3c), and 2283 proteins in UWB1.289+BRCA1 cells (Figure 3d).

**Figure 3:**
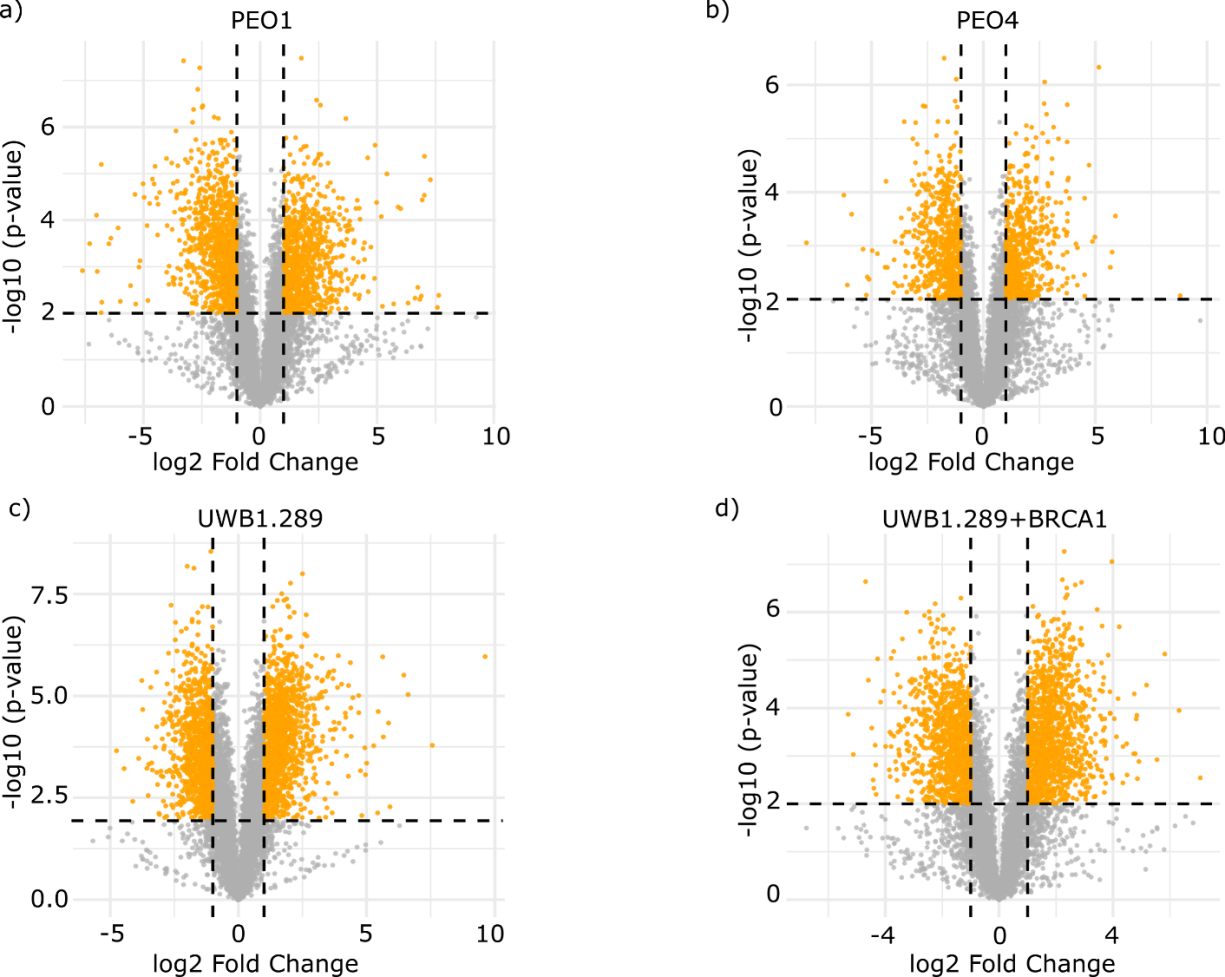
Differential regulation between 3D and 2D cultures for each cell type. Volcano plots of differentially expressed proteins depending on cell culture dimension (3D vs 2D) for (a) PEO1, (b) PEO4, (c) UWB1.289, and (d) UWB1.289+BRCA1 cells. Data filtering with a *p*-value threshold of 0.01 and an absolute log_2_ fold change >1 was applied. Orange points represent proteins that are significantly upregulated (positive log_2_ fold change) or downregulated (negative log_2_ fold change) in 3D models, while proteins that did not reach statistical significance are represented as gray points.

This analysis reveals a widespread effect of culture dimension in the proteomic landscape of the cell lines, with PEO4 cells exhibiting fewer changes than the other cell lines (Figure 3b). We then performed gene set enrichment analysis for each cell line using the ranked log_2_ (3D/2D) values and visualized significant GO terms on ridgeline plots showing the direction of change. We show that, as reported for other cancer types^15,20^ and as suggested from the overall differential analysis (Figure 2b), 3D models display significantly higher abundances of proteins involved in energy metabolism related to the mitochondria (Figures S2 and S3) than cells cultured as 2D monolayers.

While PEO4 cells seem to be less impacted by culture dimension (Figure 3b), PEO1 and UWBs cells display a more pronounced downregulation of proteins associated with the actin cytoskeleton and stress fibers (Figures S2 and S3). We hypothesize that these variations are related to changes in the cellular shape and mechanical support in cells cultured in 3D scaffold-based matrices. Network analysis of each 3D vs 2D comparison (Figures S4 and S5) highlights the relationships and shared proteins between mitochondrial-associated and other enrichment terms. Many proteins from the NDUF protein class are mostly upregulated in spheroids (Figure S6a). These proteins are part of the NADH:ubiquinone oxidoreductase (Complex I) in the mitochondrial electron transport chain^28,29^ and involved in oxidative phosphorylation. We found that ATP6AP1 and ATP1B1 are consistently upregulated in 3D models (Figure S6b).

Unlike most studies that compare 3D and 2D culture dimensions using a single cell line model, we then focused our analysis on proteins consistently up- or downregulated across all four cell lines in the same direction, aiming to identify a universal protein signature of 3D cell culture. We found 371 proteins that are differentially regulated (absolute log_2_ ratio > 1, *p*-value < 0.01) and detected across all four cell lines. Notably, among these proteins, 166 are unidirectionally upregulated and 200 downregulated, while only 5 are differentially regulated depending on the cell type.

We then performed GO analysis on the 366 proteins with common significant differential regulation across all cell lines to identify enriched molecular functions. This analysis demonstrates upregulation and enrichment in proteins mainly related to transmembrane transporter activities, ion transport, and oxidoreductase activity (Figure 4a).

**Figure 4:**
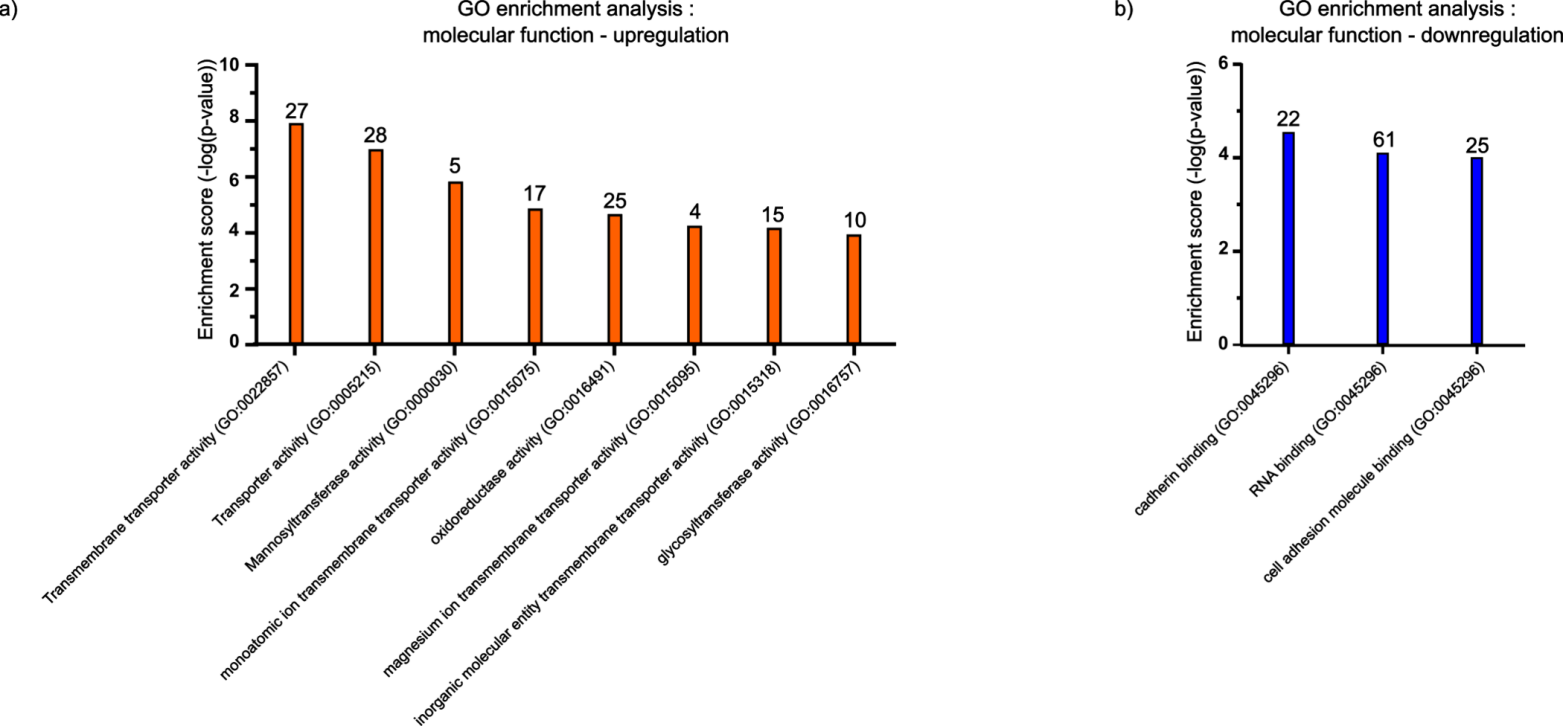
Commonly regulated biological processes in 3D versus 2D across the cell types. GO enrichment analysis for molecular function that are (a) upregulated and (b) downregulated. Analysis is performed on the 366 proteins that are consistently and significantly differentially regulated (absolute log_2_ ratio > 1, *p*-value < 0.01) among PEO1, PEO4, UWB1.289, and UWB1.289+BRCA1 cells. Numbers indicate how many proteins contribute to each term.

For example, proteins such as CNNM4 ^30^ and ANO10 ^31^ are associated with ion transmembrane transport (Figure 4a) and are upregulated (Figure S7a) across all spheroid models. Downregulated proteins are mainly associated with gene regulation.^15^ including cadherin binding and RNA binding, as reported in the literature for other cancer models (Figure 4b). We also found enrichment in downregulated proteins associated with cadherin binding (Figure 4b), a term associated with proteins that mediate calcium-dependent cell–cell adhesion. Associated downregulated proteins include SPTBN1 ^32^ and CALM3 ^19^ (Figure S7b) that are involved in cell shape endocytosis, stabilization of cell junctions, and cytoskeletal organization at the plasma membrane. These results indicate remodeling of the cell architecture in spheroids cultured in scaffold-based matrices.

### Culture Dimension Effects Include Key Membrane-Associated Proteins in 3D HGSOC Models

With many proteins known to display membrane localization, related to cell shape, membrane trafficking, and ion transport (Figures 4 and S7), we next questioned how the cell dimension influences the composition of the membrane-related proteins of HGSOC cells. Similarly to the analyses of the differentially expressed proteins in each cell line grown in 3D and 2D (Figure 3), we annotated membrane proteins among proteins that were differentially expressed between 3D and 2D in at least one cell line, with a more relaxed *p*-value threshold of 0.05 and an absolute log_2_ fold change higher than 1. Under these conditions, we found 2342 proteins differentially regulated between 3D and 2D PEO1 cells, 1892 proteins in PEO4 cells, and 1279 proteins commonly changing with cell dimension between PEO1 and PEO4 cells (summary in Table 1).

**Table 1:**
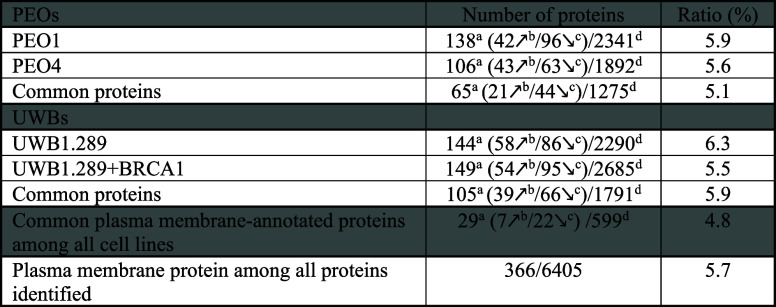
Variations of Plasma Membrane-Annotated Proteins across All Cell Lines Grown in 3D and 2D

a Plasma membrane-annotated proteins.

b,c Upregulated and downregulated plasma membrane proteins.

d Total number of proteins differentially regulated between 3D and 2D models. Data filtering is performed with a *p*-value threshold of 0.05 and an absolute log_2_ fold change higher than 1.

Regarding UWB cells, we found 2,290 differentially regulated proteins between UWB1.289 cells grown in 2D and 3D, 2685 proteins in UWB1.289+BRCA1 cells, and 1791 proteins common to both cell lines (summary in Table 1). By calculating the ratios of proteins that have a plasma membrane annotation from the Human Protein Atlas database, calculated as the number of plasma membrane-annotated proteins divided by the total number of differentially regulated proteins between 3D and 2D samples for each cell line, we found no enrichment in plasma membrane-annotated proteins. Specifically, with 366 plasma membrane-annotated proteins among 6405 identified proteins (5.7%), the plasma membrane-related proteins have similar ratio % and therefore are not particularly more impacted by culture dimension (Table 1).

Nonetheless, this analysis reveals the differential expression of key plasma membrane proteins. It also highlights that differentially regulated plasma membrane proteins tend to be generally downregulated in spheroids compared to 2D models (Table 1 and Supplementary file S4). To gain deeper insight into the specific changes of the membrane-related proteins depending on the culture dimension, we report in bidirectional bar plots the 15 most differentially up- and downregulated plasma membrane-annotated proteins for each cell line (Figure 5).

**Figure 5:**
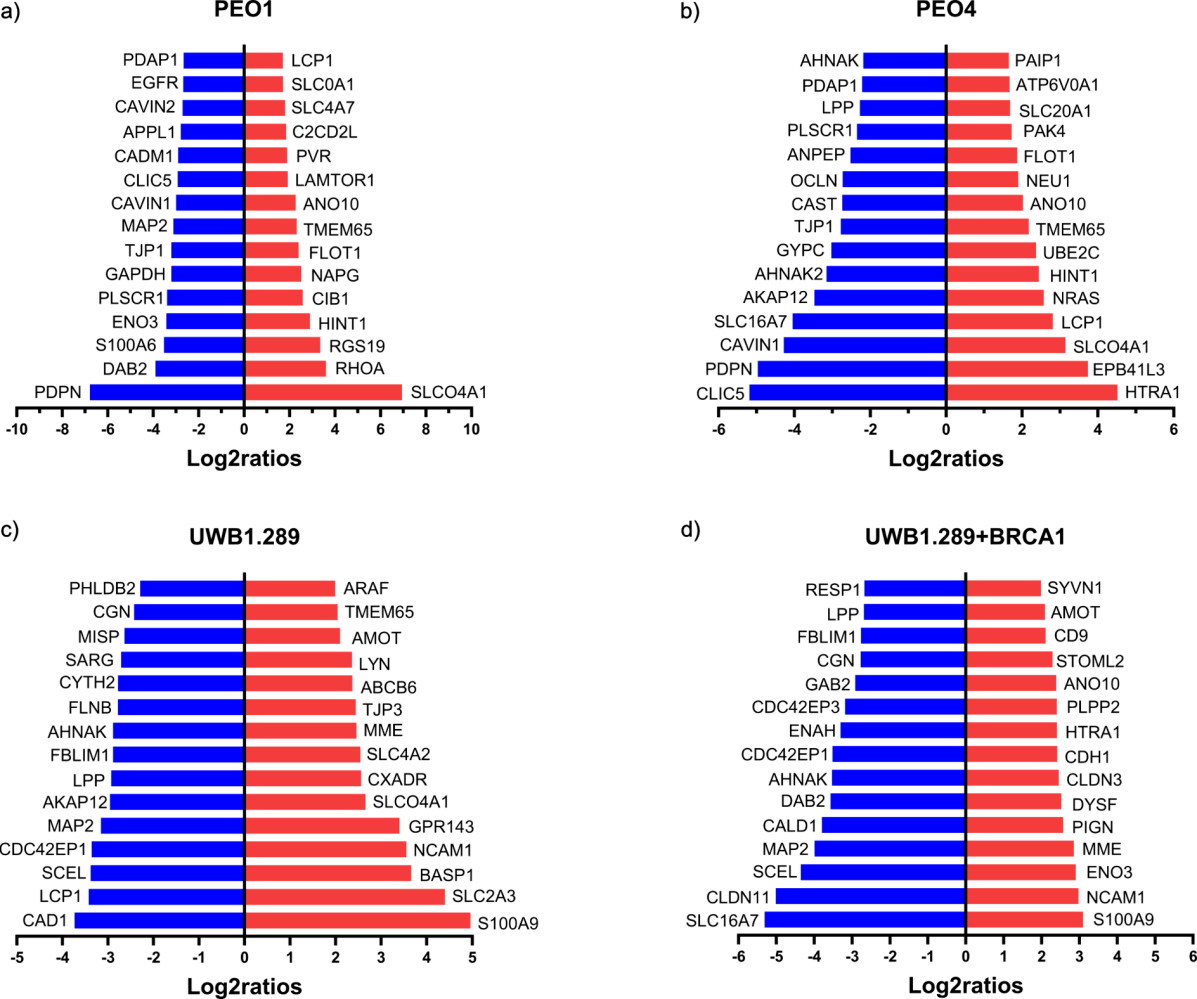
Top 15 most differentially expressed plasma membrane-annotated proteins (absolute log_2_ fold change >1, *p*-value 0.05) between 3D and 2D cultured (a) PEO1, (b) PEO4, (c) UWB1.289, and (d) UWB1.289+BRCA1 cells.

On top of CNNM4 and ANO10 (Figure 5 and Supplementary file S4) that are commonly upregulated in all spheroids, we find changes in the expression level of key plasma membrane-associated proteins such as upregulation of RHOA, a member of the Rho family of small GTPas involved in the Akt pathway, in PEO1 spheroids (Figure 5a and Supplementary file S5). Increased expression of these proteins may indicate changes in cell signaling in 3D spheroids. We also find upregulation of NRAS^33^ and PAK4 ^34^ in PEO4 spheroids, which are membrane-related proteins that also regulate crucial signaling pathways (MAPK/ERK, PI3K/AKT) (Figure 5b and Supplementary file S5).

Interestingly, we found EGFR, a well-known target for anticancer drugs, to be downregulated in PEO1 (log_2_ ratio 2.7) (Figure 5a) and PEO4 (log_2_ ratio 0.97) spheroids (Supplementary file S5). This downregulation was further validated by WB analysis, confirming the reduced EGFR expression in 3D cultures of both cell lines (Figure S8). In both UWB1.289 and UWB1.289+BRCA1 spheroids, common upregulated proteins including S100A9 ^35^ and NCAM1 ^36^ are proteins implicated in cancer progression and are explored as potential therapeutic targets (Figure 5c,d). SLC20A1, a sodium-phosphate cotransporter considered a potential therapeutic target in different cancer types, showed reduced expression (Supplementary file S6).

We then performed annotation of plasma membrane proteins among the 599 proteins (Table 1) that are differentially regulated (absolute log_2_ ratio > 1, *p*-value < 0.05) and detected among all 3D vs 2D models. We found 29 plasma membrane-annotated proteins that are unidirectionally and commonly differentially expressed among PEOs and UWBs 3D vs 2D models. Most plasma membrane-annotated proteins are downregulated, and among the 22 downregulated proteins, RDX, LPP, and CAST are of particular interest as they are involved in cell–cell interactions, signal transduction, and membrane dynamics (Supplementary files S4, S5, and S6). Among these 29 proteins, 7 are upregulated, including FLOT1 a novel biomarker^37^ (Supplementary file S4). ATP6 V0A2, a subunit of the heteromultimeric vacuolar ATPase (v-ATPase) enzyme, involved in cisplatin resistance in ovarian cancer and targeted by phyllanthusmin anticancer compounds,^38^ is upregulated in all 3D models (Figure 5c,d and Supplementary file S4). While ATP6 V0A2 upregulation is statistically in significant in three cell lines, it is borderline for PEO1 (*p-*value = 0.059).

To further support our findings, we performed a single-shot DIA analysis in independently grown PEO1 and UWB1.289 cells grown in 2D and 3D. This analysis demonstrated a high agreement with the overlapping TMT data overall and across the selected proteins discussed above (Supplementary file S7).

### Culture Dimension-Dependent Acquired Resistance to Carboplatin

Finally, given the culture dimension-dependent expression of CNNM4,^30^ ATP6 V0A2,^39^ ATP5F1C,^40^ and SLCO4A1,^41^ along with the upregulation of many proteins involved in the mitochondria complex I,^42,43^ each of which has been linked to drug resistance, we hypothesized that drug sensitivity in HGSOC models would yield cell culture dimension-dependent results, as described in other studies.^44,45^ We set out to measure, for each cell line, the GI_50_ of carboplatin in 3D and 2D cell cultures and found differences in sensitivity to carboplatin as a function of cell culture dimension (Figure 6).

**Figure 6:**
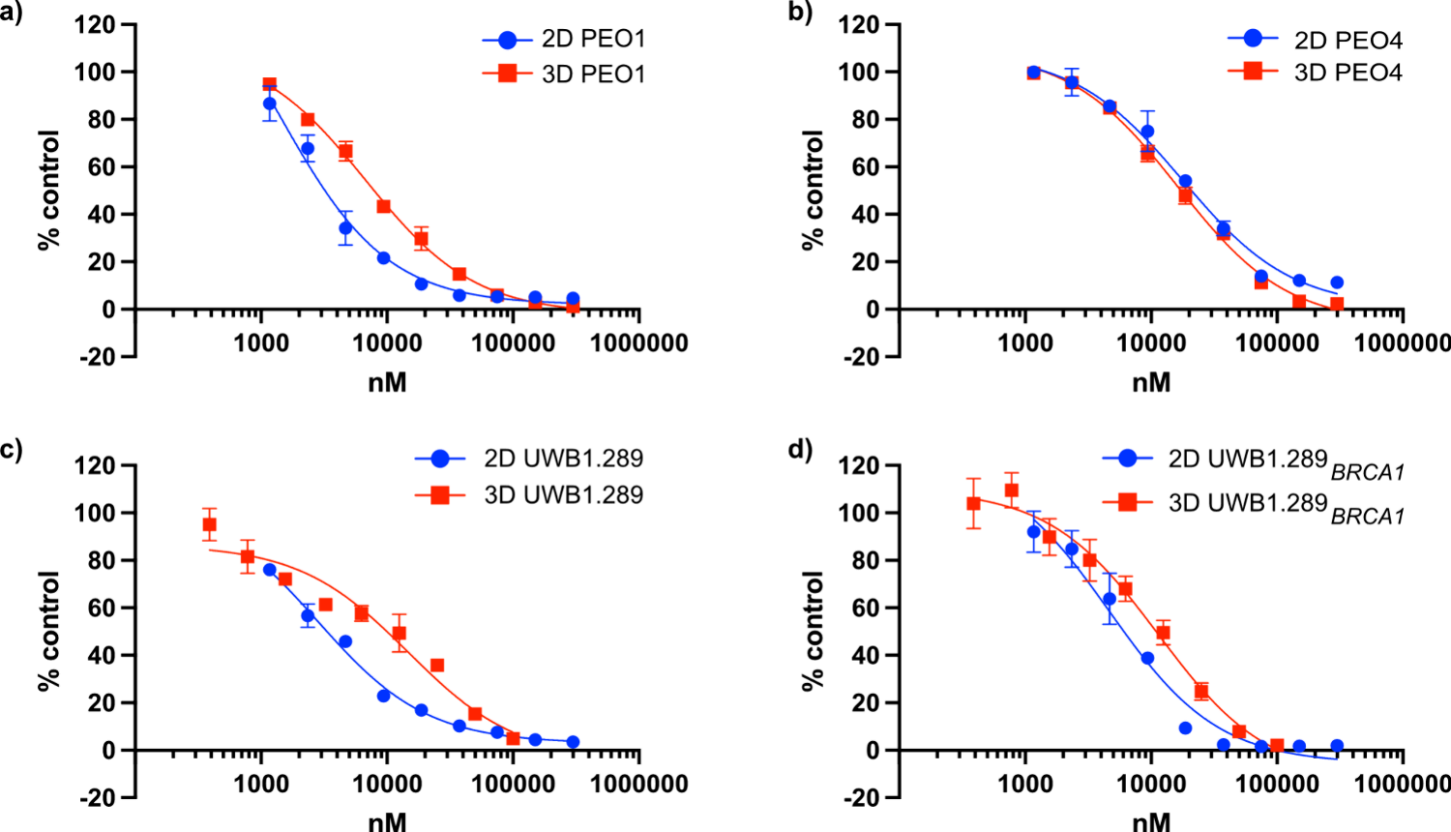
Culture dimension-dependent chemosensitivity to carboplatin of the HGSOC models. Log_10_ concentrations of drugs are plotted versus the percentage of control for (a) PEO1, (b) PEO4, (c) UWB1.289, and (d) UWB1.289+BRCA1. Carboplatin doses ranged from 0 (control) to 300 μM. Error bars represent ± the standard deviation of the average fluorescence intensity, with respect to the percentage of control, from three independent experiments. While error bars are consistently included, they may not be visible when smaller than the symbol size.

While PEO1 and UWB1.289 are more sensitive to carboplatin than PEO4 and UWB1.289+BRCA1 in 2D cultures, surprisingly, 3D samples displayed similar sensitivities, irrespective of the BRCA status of the cell lines (Figure 6 and Table S1). PEO4, a cell line less affected by culture dimension at the proteome level (Figure 3) and derived from platinum-resistant cells,^25^ is the only cell line displaying similar GI_50_ in 3D and 2D samples (Figure 6). Overall, these results highlight that many proteins associated with drug resistance are upregulated in spheroids. This differential cell culture-dependent regulation correlates with the observed differences in GI_50_ values between 2D and 3D models, demonstrating increased and culture dimension-dependent acquired resistance to carboplatin in 3D models.

## Discussion

Consistent with previously published studies on other cancer types,^15,20^ we found significant variation in protein expression levels between 2D and 3D cultured HGSOC cells. Our data show that while genotype significantly influenced the proteomic profiles, the culture dimension introduces profound changes in the expression of proteins associated with key cellular processes. As demonstrated by Kerslake et al. at the transcriptome level,^22^ we observed that energy metabolism, particularly mitochondrial function, was significantly enriched in the 3D culture conditions. However, unlike the transcriptomic findings, we did not find, at the proteome level, significant changes in glycolysis. Proteins of the mitochondrial electron transport chain such as ATP5F1C and proteins from the NDUF family were particularly upregulated in our spheroid models. This aligns with the hypothesis that 3D models provide a more metabolically active and oxygen-dependent environment and may better mimic in vivo conditions.^46^

We also identified significant changes in proteins involved in cellular signaling pathways and related to structural components such as the actin cytoskeleton, suggesting the reorganization of the cytoskeleton in 3D HGSOC models.

We found significant changes in the regulation of several membrane-bond proteins, despite the identification of no particular culture dimension-dependent enrichment in plasma membrane-annotated proteins. We found significant downregulation of EGFR in both PEO1 and PEO4 spheroids and unidirectional and upregulation of TMEM65, SLCO4A1, CNNM4, ANO10, and FLOT1 across all 3D samples. The downregulation of several proteins, such as RDX, LPP, and CAST, further supports the idea that 3D culture induces some changes at the plasma membrane.

Consistent with the upregulation of GPNMB and CHI3L1 in 3D compared to 2D cultures of patient-derived ovarian models reported by Franciosa et al.,^47^ we also observed increased expression of these proteins specifically in UWB1.289 cells grown in 3D. However, this upregulation was not conserved across all other ovarian cancer cell lines analyzed.

Interestingly, PEO4 cells appeared to be less influenced by the culture dimension and displayed a similar sensitivity to carboplatin in 3D and 2D cell cultures. Although PEO1, PEO4 cells and UWB1.289, UWB1.289+BRCA1 represent two pairs of closely related cell lines, the correlations within each pair remain relatively weak, and we only found 371 proteins that are commonly associated with cell culture dimension across all four cell models. These results emphasize that culture dimensions impact cell lines with both common and specific protein expression changes. The culture dimension-dependent proteomic features include proteins linked to drug resistance and the mitochondrial complex I.^42,43^

We hypothesize that the lack of cell-dimension-dependent sensitivity to carboplatin in PEO4 arises from genetic alterations rather than a culture dimension-dependent proteomic signature. Indeed, the cell line was derived after the patient had developed resistance to platinum-based chemotherapy,^25^ suggesting the cells may have reached a “plateau” in terms of resistance and that further exposure to the 3D culture system does not lead to additional resistance. While it has been described that drug diffusion is reduced in 3D settings and can lead to increased resistance,^48^ our findings further suggest that proteomic markers of cell culture dimension also influence the response of cells to carboplatin in spheroids, thus potentially overriding the genetic differences observed in 2D cultures, as already observed at the transcriptome level for ovarian cancer cells.^44^

While our analysis provides a comprehensive overview of proteomic alterations in response to culture dimensionality, the functional implications of these changes remain to be further validated through functional assays. In particular, our approach does not selectively enrich cell-surface proteins and instead focuses on global proteomic changes including membrane-associated proteins broadly defined by annotation.

## Conclusions

Unlike most previous studies that compared the impact of cell culture dimensionality in unique sets of cell lines grown in 3D and 2D, here, we use two pairs of HGSOC cell lines, allowing us to investigate the degree of similarity or differences in the proteome associated with culture dimensionality rather than similarities in their genomics. Our findings suggest that HGSOC spheroid models provide more accurate insights into drug response and better reproduce in vivo*-like* metabolism than cell monolayers. In contrast, 2D cell cultures are likely relevant models for membrane-related drug target discovery, such as for the development of immunotherapies, as we did not observe the enrichment of such proteins in spheroids. Hence, the choice between 3D and 2D models in ovarian cancer research should be tailored to specific applications and should be driven by the specific research question. Furthermore, the comparison of spheroids to patient-derived organoids remains to be studied to evaluate the use of spheroids as improved models for ovarian cancer research.

## Supplementary Material

















## Data Availability

Mass spectrometry proteomics data have been deposited to the ProteomeXchange Consortium via the PRIDE^49^ partner repository with the data set identifiers PXD062934 and PXD066652.

## Notes

PCA plot generated prior to reference-based normalization mean across samples; gene set enrichment analysis (ridge plot) of PEO cells grown in 3D vs 2D; gene set enrichment analysis (ridge plot) of UWB cells grown in 3D vs 2D; gene set enrichment analysis (cumulative network enrichment) of PEO cells grown in 3D vs 2D; gene set enrichment analysis (cumulative network enrichment) of UWB cells grown in 3D vs 2D; heatmap representation from selected gene set enrichment analysis significant terms; heatmap representation from selected gene set enrichment analysis significant terms; WB analysis of EGFR expression in 3D vs 2D cultures of PEO1 and PEO4 cells; and growth inhibition measurements of carboplatin in HGSOC cell lines cultured in 2D and 3D (PDF)
TMT-labeling scheme for multiplexed proteomic analysis (XLSX)
ANOVA analysis of protein expression across 2D vs 3D cultures in HGSOC cell lines (XLSX)
Differential protein expression (log_2_ fold changes) between 3D and 2D cultured HGSOC cell lines (XLSX)
List of common membrane-annotated proteins differentially expressed between 3D and 2D cultured HGSOC cell lines (XLSX)
List of commonly up- and downregulated proteins in PEO1 and PEO4 cell lines grown in 3D vs 2D cultures (XLSX)
List of commonly up- and downregulated proteins in UWB1.289 and UWB1.289+BRCA1 cell lines grown in 3D vs 2D cultures (XLSX)
DIA-based verification of DDA-identified protein expression changes in UWB1.289 and PEO1 cell lines cultured in 3D and 2D­(XLSX)

## ABBREVIATIONS

2D: 2-Dimensional
3D: 3-Dimensional
DIA: Data-Independent Acquisition
GI: Growth Inhibition
GO: Gene Onotology
HGSOC: High-grade Serous Ovarian Carcinoma
hEGF: human Epidermal Growth Factor
LC: Liquid Chromatography
MEM: Minimum Essential Medium
MS: Mass Spectrometry
NF-κB: Nuclear factor-kappa B
PEOs: PEO1 and PEO4
RPMI: Roswell Park Memorial Institute
MS/MS: Tandem Mass Spectrometry
TMT: Tandem Mass Tag
TNF-α: Tumor Necrosis Factor Alpha
UWBs: UWB1.289 and UWB1.289+BRCA1
WB: Western Blot
