# Supplementary material for: Proteomic Landscapes of 3D and 2D Models of High-Grade Serous Ovarian Carcinoma: Implications for Carboplatin Response
Source: J Proteome Res. 2025 Aug 27;24(10):5071–82. doi: 10.1021/acs.jproteome.5c00391 (PMC12501941; doi:10.1021/acs.jproteome.5c00391)
Supplement: Supplementary file 1 [file pr5c00391_si_001.pdf]

Supporting Information

Proteomic landscapes of 3D and 2D models of High-Grade Serous Ovarian Carcinoma: Implications for Carboplatin Response

Jimmy Maillard[1], Theodoros I. Roumeliotis[2], Ekta Paranjape[1], Lisa Pickard[1], Alvaro Ingles R. Garces,[1] Jyoti S. Choudhary[2] and Udai Banerji[1,3]**

[1]Clinical Pharmacology Adaptive Therapy group. Division of Clinical Studies and Division of Cancer Therapeutics, Institute of Cancer Research, London, SM2 5NG, United Kingdom, [2]Functional Proteomics group, Chester Beatty Laboratories, The Institute of Cancer Research, London, SW3 6JB, UK, [3]The Drug Development Unit, The Institute of Cancer Research and The Royal Marsden Hospital NHS Foundation Trust, London, SM2 5NG, United Kingdom

*Correspondence: jyoti.choudhary@icr.ac.uk, udai.banerji@icr.ac.uk

Table of supplementary contents

Figure S1..3
Figure S2. 4
Figure S3. 5
Figure S4. 6
Figure S5. 7
Figure S6. 8
Figure S7..9
Figure S8..11
Table S1. 11
Supplementary File S1. TMT-labelling scheme for multiplexed proteomic analysis……………...
Supplementary File S2. ANOVA analysis of protein expression across 2D vs 3D cultures in HGSOC cell lines………………………………………………………………………...
Supplementary File S3. Differential Protein Expression ($\log_2$ Fold Changes) between 3D vs 2D cultured HGSOC cell lines…………………………………………………………………….
Supplementary File S4. List of common membrane-annotated proteins differentially expressed between 3D vs 2D cultured HGSOC cell lines……………………………………………………
Supplementary File S5. List of commonly up- and downregulated proteins in PEO1 and PEO4 cell lines grown in 3D vs 2D Cultures……………………………………………………………
Supplementary File S6. List of commonly up- and downregulated proteins in UWB1.289 and UWB1.289+BRCA1 cell lines grown in 3D vs 2D Cultures……………………………………..
Supplementary File S7. DIA-based verification of DDA-identified protein expression changes in UWB1.289 and PEO1 cell lines cultured in 3D and 2D……………………………………………..

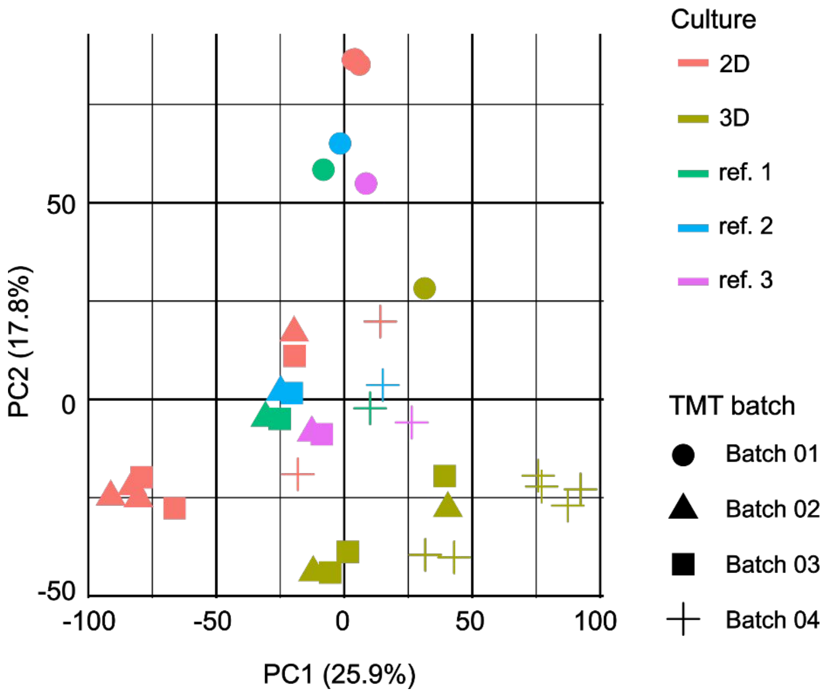

Figure S1. PCA plot generated from median-normalized and log_2-centered data across all samples prior to reference-based normalization mean across samples to evaluate batch effects.

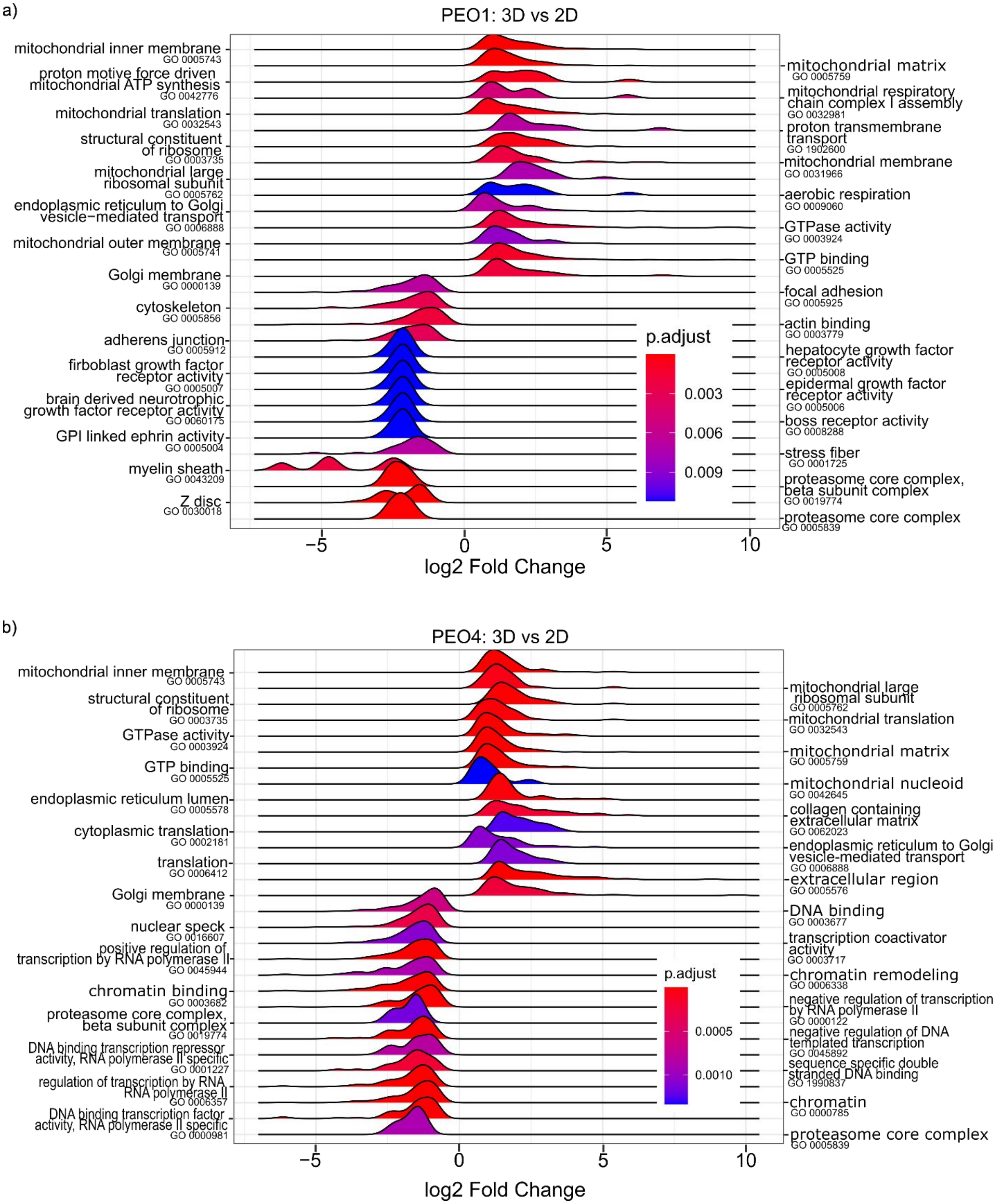

Figure S2. Gene set enrichment analysis represented on ridge plots for a) PEO1 and b) PEO4 models grown in 3D vs 2D showing distribution of leading-edge log2(3D/2D).

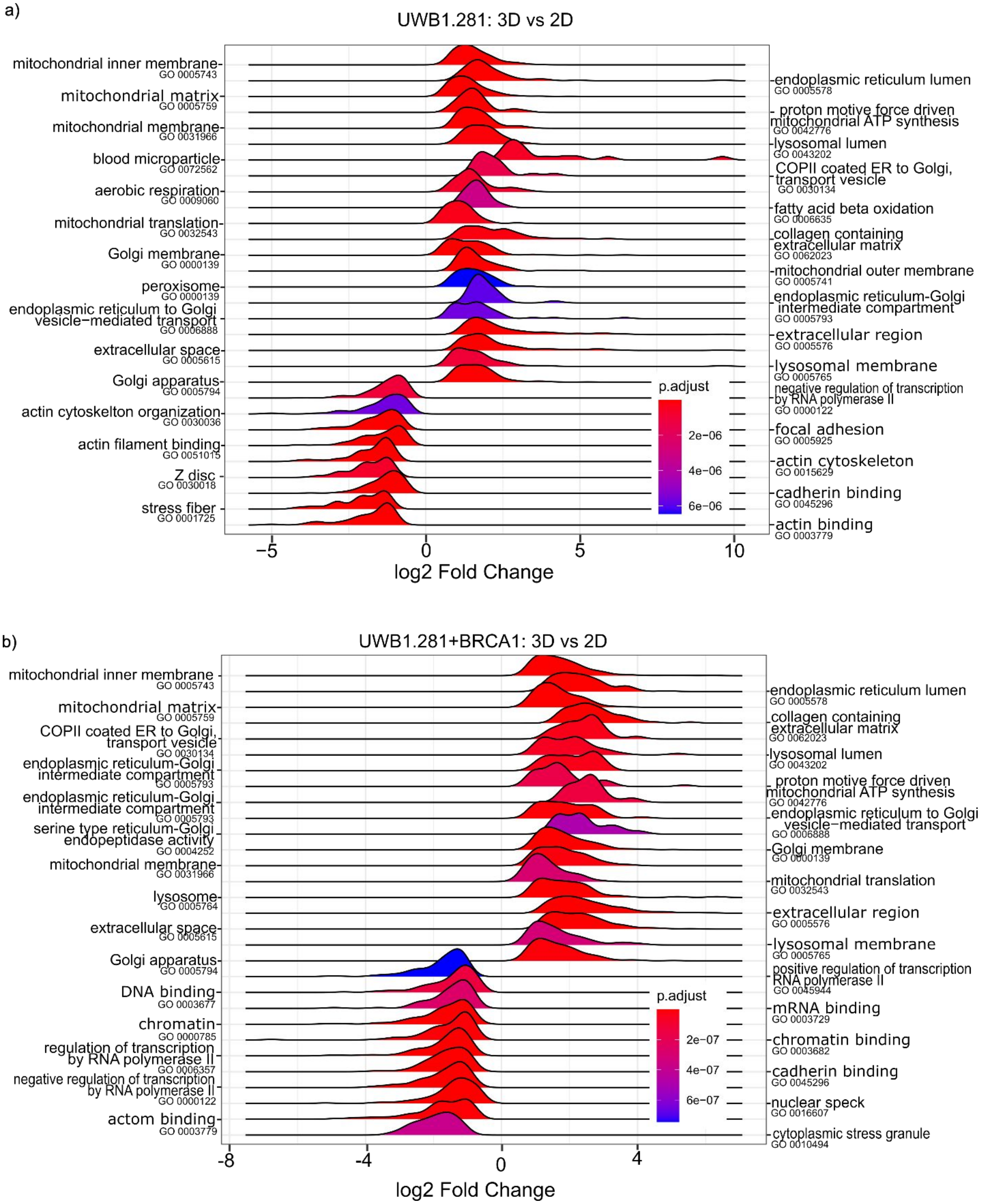

Figure S3. Gene set enrichment analysis represented on ridge plots for a) UWB1.289 and b) UWB1.289+BRCA1 models grown in 3D vs 2D showing distribution of leading-edge log2(3D/2D).

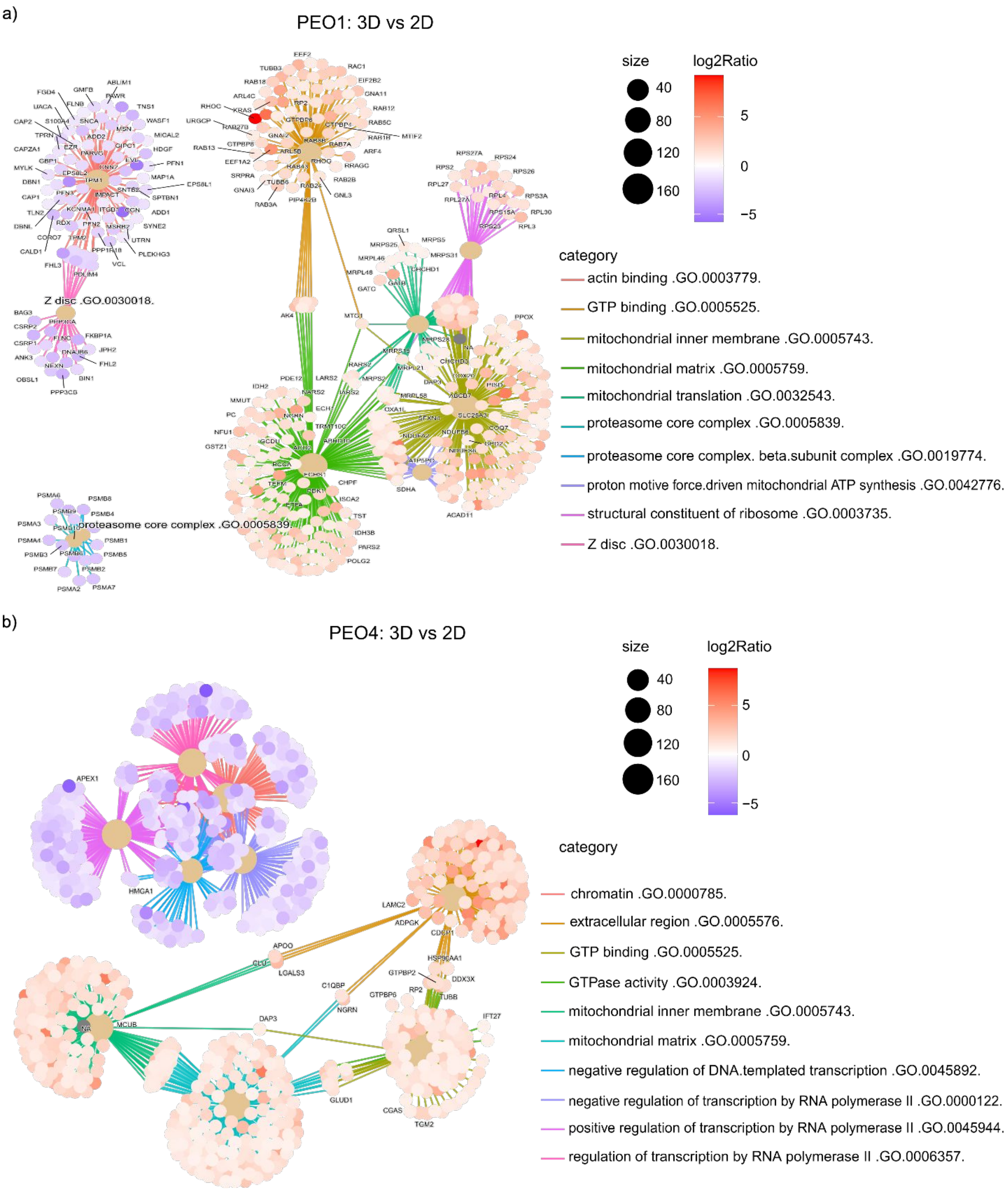

Figure S4. Results from gene set enrichment analysis (p.adjust<0.05) represented on concept network enrichment plots for leading-edges with strongest differential regulation among 3D and 2D models of a) PEO1 and b) PEO4 cells.

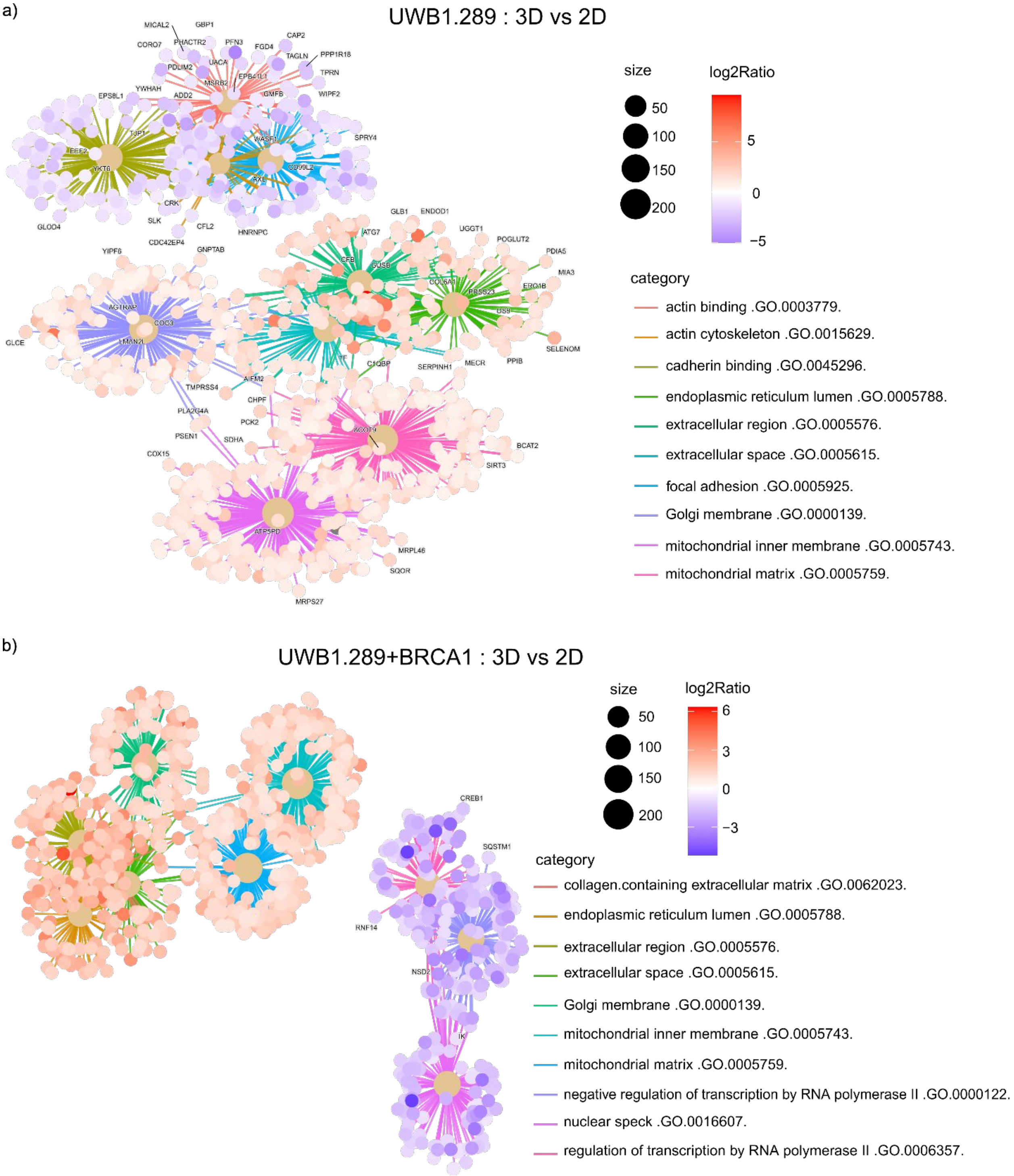

Figure S5. Results from gene set enrichment analysis (p.adjust<0.05) represented on concept network enrichment plots for leading-edges with strongest differential regulation among 3D and 2D models of a) UWB1.289 and b) UWB1.289+BRCA1 cells.

a)

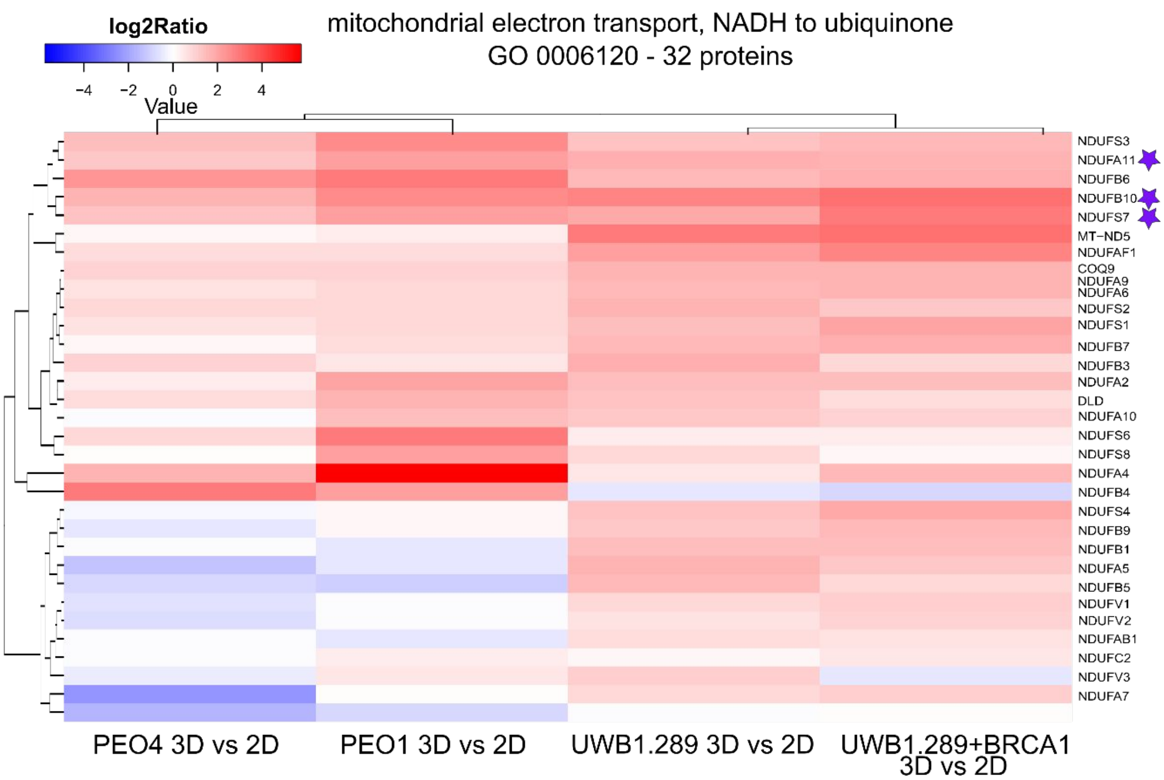

b)

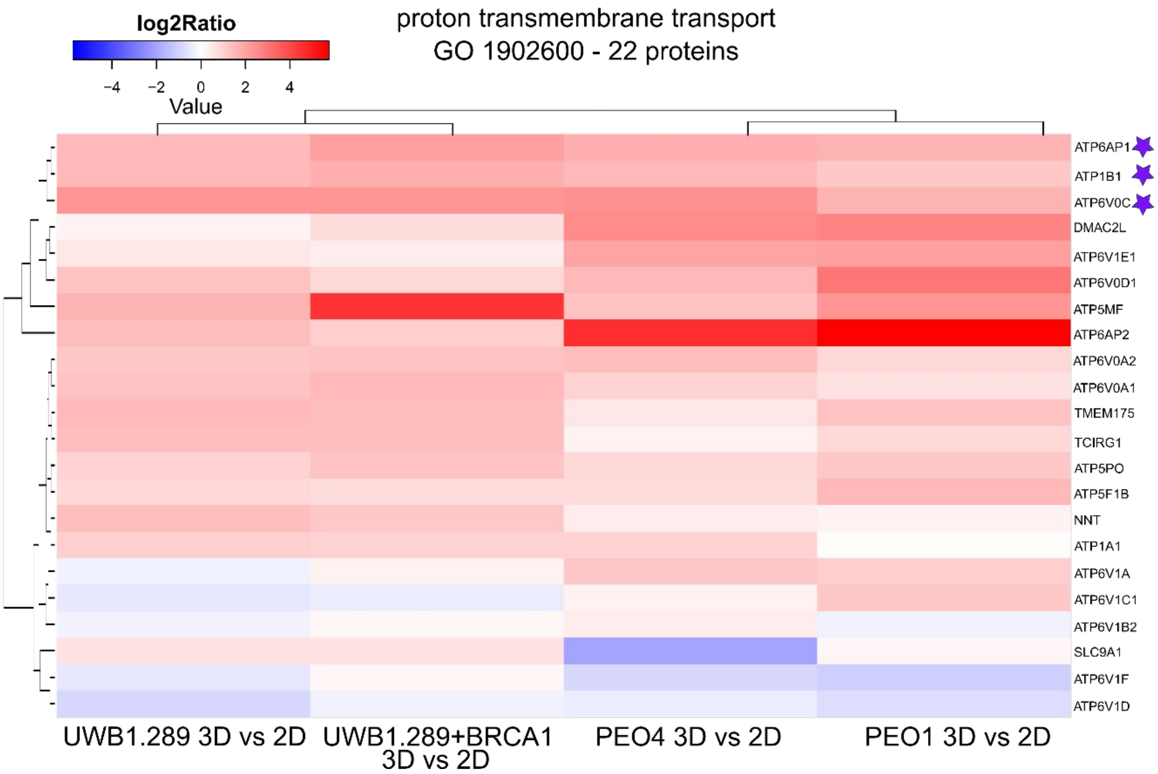

Figure S6. Heatmap representation of proteins from selected gene set enrichment analysis significant terms (p.adjust<0.05) for a) mitochondrial electron transport, NADH to ubiquinone and b) proton transmembrane transport. Proteins marked with a purple star belong to the 366 proteins that were differentially regulated (absolute log2 ratio>1, p-value <0.01) in all spheroids.

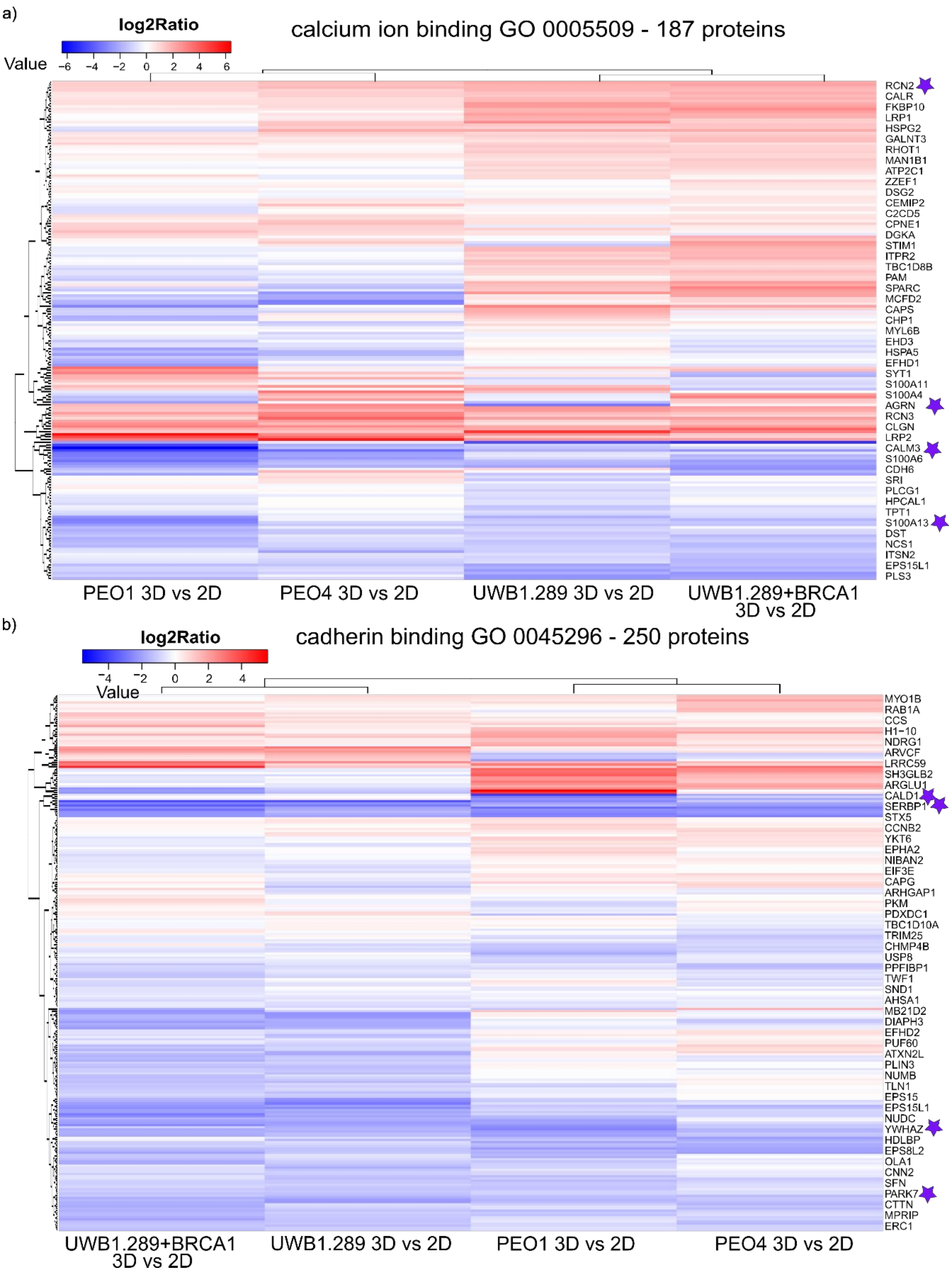

Figure S7. Heatmap representation of proteins from selected gene set enrichment analysis significant terms (p.adjust<0.05) for a) calcium ion binding and b) cadherin binding terms. Proteins

marked with a red star belong to the 366 proteins that were differentially regulated (absolute log2 ratio>1, p-value <0.01) in all spheroids.

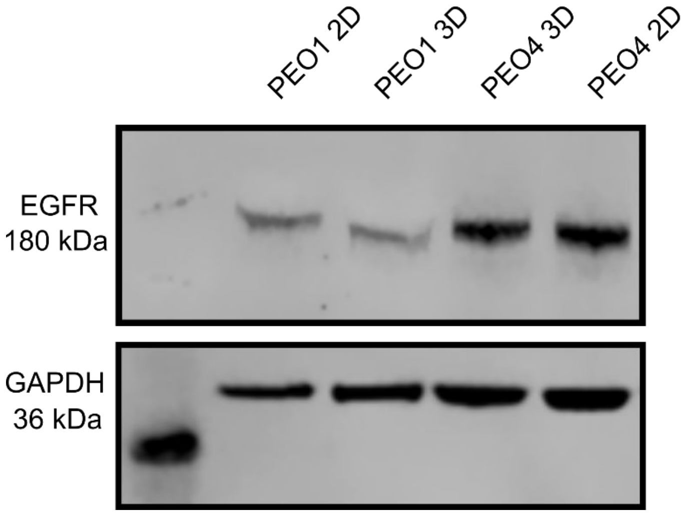

Figure S8. Western blot analysis confirms EGFR expression is reduced in 3D-cultured PEO1 and PEO4 cells compared to 2D cultures.

Table S1. GI_{50} values for carbopatin were determined from 3 independent experiments. Each cell type, grown in 2D and 3D, was exposed to increasing doses of carboplatin (0-300 μM) for 5 days.

Cell type & culture dimension	GI_{50} (nM)			Mean (nM)	Standard deviation	Standard error of the mean
2D UWB1.289	934.8	938.8	601.3	825.0	193.7	112.0
3D UWB1.289	10813	11587	8785	10395.0	1447.0	836.4
2D UWB1.289+BRCA1	4636	1702	1703	2680.3	1693.7	979.0
3D UWB1.289+BRCA1	11727	11312	9122	10720.3	1399.7	809.1
PE01 2D	2447	1354	2075	1958.7	555.7	321.2
PE01 3D	13291	12869	6765	10975.0	3652.1	2111.0
PE04 2D	16389	17733	14343	16155.0	1707.1	986.7
PE04 3D	10292	16330	6075	10899.0	5154.4	2979.4
